# Micronutrient Deficiencies and *Plasmodium vivax* Malaria among Children in the Brazilian Amazon

**DOI:** 10.1371/journal.pone.0151019

**Published:** 2016-03-10

**Authors:** Silvana Gomes Benzecry, Márcia Almeida Alexandre, Sheila Vítor-Silva, Jorge Luis Salinas, Gisely Cardoso de Melo, Helyde Albuquerque Marinho, Ângela Tavares Paes, André Machado de Siqueira, Wuelton Marcelo Monteiro, Marcus Vinícius Guimarães Lacerda, Heitor Pons Leite

**Affiliations:** 1 Escola Superior de Ciências da Saúde, Universidade do Estado do Amazonas, Manaus, Brazil; 2 Gerência de Malária, Fundação de Medicina Tropical Dr. Heitor Vieira Dourado, Manaus, Brazil; 3 Division of Infectious Diseases, Department of Medicine, Emory University, Atlanta, United States of America; 4 Laboratório de Nutrição, Instituto Nacional de Pesquisas da Amazônia, Manaus, Brazil; 5 Applied Statistics, Federal University of São Paulo, São Paulo, Brazil; 6 Instituto Nacional de Infectologia Evandro Chagas, Rio de Janeiro, Brazil; 7 Instituto de Pesquisas Leônidas & Maria Deane, Fundação Oswaldo Cruz, Manaus, Brazil; 8 Department of Pediatrics, Discipline of Nutrition and Metabolism, Federal University of São Paulo, São Paulo, Brazil; Université Pierre et Marie Curie, FRANCE

## Abstract

**Background:**

There is a growing body of evidence linking micronutrient deficiencies and malaria incidence arising mostly from *P*. *falciparum* endemic areas. We assessed the impact of micronutrient deficiencies on malaria incidence and *vice versa* in the Brazilian state of Amazonas.

**Methodology/Principal Findings:**

We evaluated children <10 years old living in rural communities in the state of Amazonas, Brazil, from May 2010 to May 2011. All children were assessed for sociodemographic, anthropometric and laboratory parameters, including vitamin A, beta-carotene, zinc and iron serum levels at the beginning of the study (May 2010) and one year later (May 2011). Children were followed in between using passive surveillance for detection of symptomatic malaria. Those living in the study area at the completion of the observation period were reassessed for micronutrient levels. Univariate Cox-proportional Hazards models were used to assess whether micronutrient deficiencies had an impact on time to first *P*. *vivax* malaria episode. We included 95 children median age 4.8 years (interquartile range [IQR]: 2.3–6.6), mostly males (60.0%) and with high maternal illiteracy (72.6%). Vitamin A deficiencies were found in 36% of children, beta-carotene deficiency in 63%, zinc deficiency in 61% and iron deficiency in 51%. Most children (80%) had at least one intestinal parasite. During follow-up, 16 cases of vivax malaria were diagnosed amongst 13 individuals. Micronutrient deficiencies were not associated with increased malaria incidence: vitamin A deficiency [Hazard ratio (HR): 1.51; *P*-value: 0.45]; beta-carotene [HR: 0.47; *P*-value: 0.19]; zinc [HR: 1.41; *P*-value: 0.57] and iron [HR: 2.31; *P*-value: 0.16]). Upon reevaluation, children with al least one episode of malaria did not present significant changes in micronutrient levels.

**Conclusion:**

Micronutrient serum levels were not associated with a higher malaria incidence nor the malaria episode influenced micronutrient levels. Future studies targeting larger populations to assess micronutrients levels in *P*. *vivax* endemic areas are warranted in order to validate these results.

## Introduction

Malaria is one of the most serious public health problems in the world, with 3.3 billion people at risk of contracting the disease and almost one million deaths annually, primarily caused by *Plasmodium falciparum* in African children under five years of age [1]. In Latin America most cases occur in the Brazilian Amazon with nearly 150,000 cases reported yearly, the vast majority caused by *P*. *vivax* (87.8%) [2].

Micronutrient deficiencies are bidirectionally associated with several infections due to specific micronutrients playing key roles in the function of the immune system. Certain deficiencies may also predispose to malaria incidence [3–6], while malaria itself has been associated with malnutrition [7–9] and possibly micronutrient deficiencies [10]. The relationship between micronutrient deficiencies and malaria comes mostly from animal [11–16] and cross sectional studies [17] predominantly in *P*. *falciparum* malaria. These data prompted several authors to propose micronutrient supplementation, i.e. vitamin A and zinc to prevent malaria incidence [5, 17, 18], severity [19] and mortality [4–6, 20, 21], an approach that was shown to be generally successful. The association between iron deficiency and malaria incidence is still controversial. Some studies have shown that iron supplementation may increase the risk of malaria incidence [22–25].

Despite the growing body of evidence associating micronutrient deficiency and *P*. *falciparum* malaria, there is still a lack of studies addressing this possible association in *P*. *vivax*. *Plasmodium vivax* is different from *P*. *falciparum* as it can lead to relapses and persist as an asymptomatic infection. Therefore, *P*. *vivax* could be associated with chronic inflammation, similar to other infections [26–31], which may have a detrimental effect on micronutrient levels as has been shown for chronic infections such as HIV [28, 31] and tuberculosis [32].

Previous studies in the Brazilian Amazon show insufficient micronutrient intake in native populations in spite of the high micronutrient content of local fruits and fish [33, 34]. We set up a prospective study to assess whether vitamin A, beta-carotene, zinc, or iron serum levels were associated with an increased risk of vivax malaria incidence, and whether having malaria affected the serum levels of these micronutrients.

## Methods

### Ethics statement

This study was approved by the Ethics Committee Board of the Fundação de Medicina Tropical Dr. Heitor Vieira Dourado (1899/2008 and 918/2010 approvals). Each participant and his/her parents or legal guardians signed written informed consent forms, after they were clear about the purpose and the study protocol.

### Study area and population

The study was undertaken in two rural communities located in recently colonized areas devoted to agriculture (Panelão and Castanho Sítio Communities), located in the Municipality of Careiro in the Amazonas State, from May 2010 to May 2011. The municipality has an area of 6,124,300 km^2^ and has 31,063 inhabitants. The climate is tropical and humid, with rainfalls ranging from 2,100 to 2,400 mm per annum. The municipality is connected to the capital of the state, Manaus, through a federal road (112 km of distance). Malaria is endemic in this area. The major economic activities are family farming, hunting and fishing. Drinking water comes from rainwater reservoirs or creeks. Garbage collection and sanitation are absent. Two health agents in each community are responsible for health care. According to a census performed in 2008 the total population of both communities was 790 people, including 300 children ranging from 1 month to 14 years of age [2, 7].

### Study design

**Fig 1:**
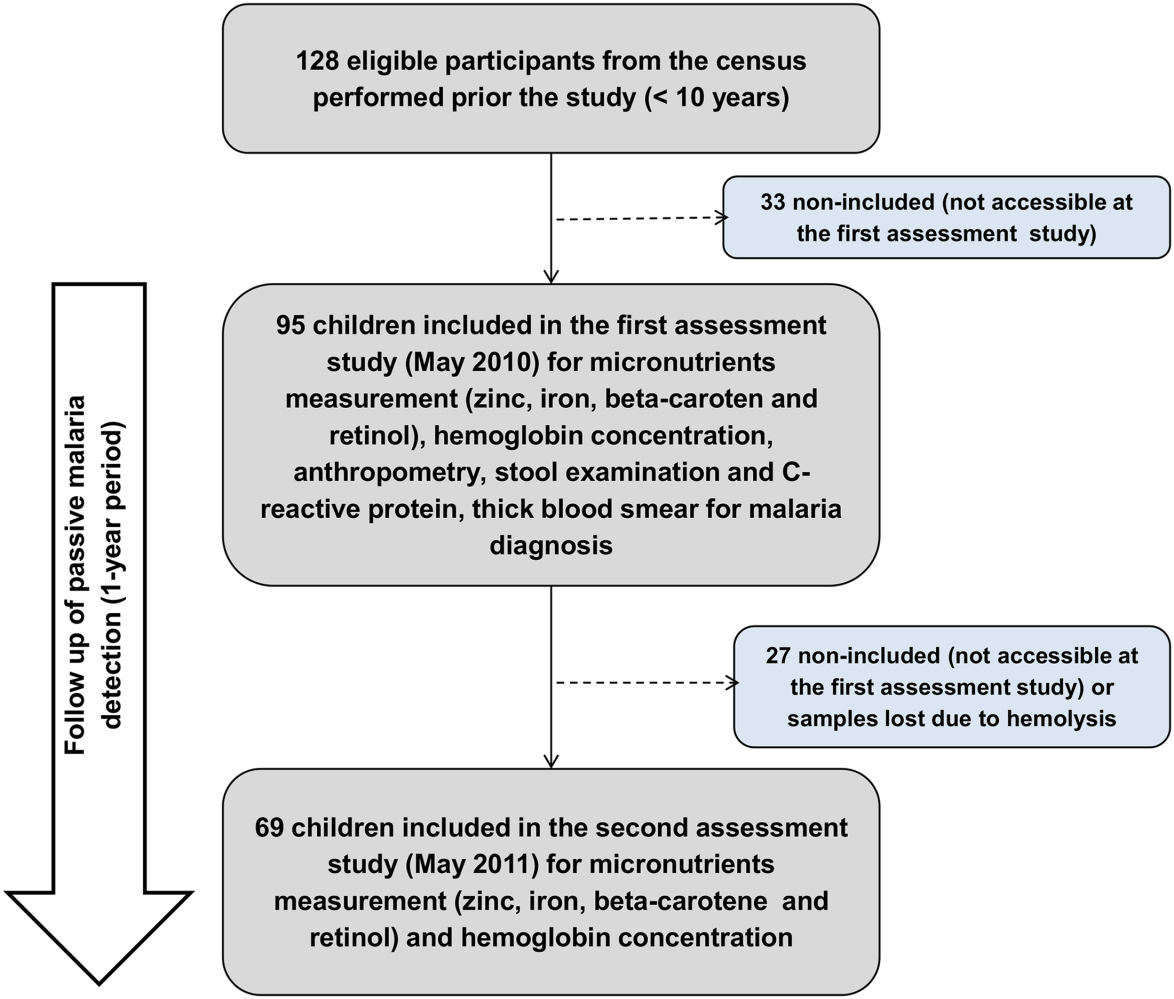
Study flow chart. From 128 eligible participants from the 2008 census, 95 children were included in the baseline assessment (2010) and 69 completed the follow up (2011).

All children <10 years residing in the study area in May 2010 (Baseline) were eligible for the study. Socio-demographic information, including health history, maternal education and housing conditions were collected. Each child underwent an anthropometric nutritional status assessment (weight and height, brachial circumference [7]), laboratory analyses for hemoglobin, micronutrient serum levels (vitamin A, beta-carotene, zinc and iron), C reactive protein, parasitological stool analyses of feces and testing for malaria (thick smear and molecular testing). After this initial evaluation, we captured information on malaria incidence via passive surveillance for 1 year, in which all febrile episodes were tested for *Plasmodium* infection. Children present in the community by May 2011 were reevaluated for the abovementioned parameters in order to assess the effects of malaria on serum micronutrient levels (Reevaluation) (Fig 1).

### Nutritional status assessment

Anthropometric measurements were performed at baseline and at reevaluation, with minimal clothing and no shoes [35]. Weight and height were obtained by internationally recommended methods [36]. Weight was measured using a digital weight scale for children above 2 years of age and a mechanical pediatric scale for younger children, both in kilograms, while height was assessed by a single observer with the use of a portable anthropometer stadiometer with lateral scale in centimeters. All instruments were adjusted and verified after each measurement and fully calibrated every three months. The measurement techniques were harmonized according to the mentioned procedures [35–37], with all team members following the same standardized protocol. Body mass index (BMI) was calculated using the ANTHRO and ANTHRO PLUS softwares [38]. Body mass index for age Z-scores below -2 were defined as undernutrition. The classification of each individual as having adequate or inadequate BMI was made according to the World Health organization (WHO) standards for each age [39].

### Micronutrient assessment

A sample of 5 ml of blood was collected from each child and that was placed on filter paper for vitamin A and beta-carotene assessments and in a tube with heparin for zinc and iron measurements. All samples were transferred to the laboratory in cold conditions and protected from light; samples were centrifuged at 15,000 rpm for 30 seconds to obtain plasma. Vitamin A and beta-carotene were assessed using high performance liquid chromatography in plasma. Erythrocytic zinc and serum iron levels were assessed by atomic chromatography [40]. The following cut-off points were used to define micronutrient deficiency: ≤10 μg/dL for vitamin A, ≤20 μg/dL for beta-carotene, ≤70 μg/dL for zinc and ≤70 μg/dL for iron [41–45].

### Malaria diagnosis

Thick blood smear (TBS) were prepared as recommended by the Walker technique [46] and evaluated by an experienced microscopist. Microscopy results were confirmed by polymerase chain reaction, using the Kit QIAmp DNA Blood Mini Kit (Quiagen, Hilden, Germany) and amplified DNA using the Applied Biosystems 7500 Fast System. A previous study by our group showed a 97.8% concordance between TBS and PCR [7]. A malaria case was documented when the child presented with fever and a positive TBS.

### Hemoglobin concentration

Hemoglobin concentration was measured in venous blood using a portable photometer (HemoCue, Anglholm, Sweden) at the beginning and at reevaluation. Anemia was defined as hemoglobin <12 g/dL [47].

### Stool examination

Stool samples were taken at baseline (May 2010) and on revaluation (May 2011) to examine the association between helminth infection and micronutrient indicators. Samples were stored in flasks containing 10% formalin as preservative. Flasks were labeled with the patient’s name and date of collection and were kept at room temperature until stool examination at the end of the day of collection. Spontaneous sedimentation [48] and centrifugal flotation in zinc sulfate solution [49] methods were applied before the samples were analyzed with a microscope for detection of helminthes and protozoan parasites using the direct observation, Kato-Katz, and the Faust and Hoffman methods. All tests were performed and evaluated by a single experienced examiner.

### Statistical analyses

Descriptive statistics were employed to evaluate overall demographic, anthropometric and laboratory characteristics to ensure that distributional assumptions for statistical tests were met. Continuous variables were reported as median [first quartile-third quartile] and categorical variables as frequencies and percentages. Univariate Cox proportional hazard models were used to assess the impact of baseline characteristics on time to malaria incidence over the one year follow up. Chi-2 or Fisher’s exact test were performed to assess the effects of malaria incidence on the delta of micronutrients serum levels (Δ = reevaluation *minus* baseline serum levels). Migration from the area was documented and follow-up time adjusted. All analyses were performed using STATA 13 (College Station, TX).

## Results

Using results from the 2008 census, 128 children would be eligible (<10 years old) by the time of the baseline assessment (May 2010). As 33 children had moved out of the area, ninety-five children were included in the study (Table 1). Median age was 4.8 years (interquartile range (IQR) [2.3–6.6]) and most were male (60.0%). A total of 33 (34.7%) of the children had previous malaria. Height-for-age (Z score <-1) was recorded in 42.1%, height-for-age (Z score <-2) in 11.6%, BMI-for-age (Z score <-1) in 17.9% and BMI-for-age (Z score <-2) in 3,2% of the children. Mean hemoglobin was 11.5 g/dL IQR [11–12.2] and anemia prevalence was 57.4%. Vitamin A deficiency was observed in 35.9%, beta-carotene deficiency in 63.0%, zinc deficiency in 60.9% and iron deficiency in 51.1% of the children. A total of 76 (80.0%) of the children had one ore more intestinal parasite. The most common intestinal parasite found was *Ascaris lumbricoides*, in 44.2% of the children.

**Table 1:** Baseline characteristics of 95 children evaluated for nutritional and micronutrient status in Careiro, Brazil, May 2010.

Baseline characteristics	Median (IQR) or N (%)
**Age**	4.8 IQR [2.3–6.6]
**Males**	57 (60.0%)
**Maternal educational level**	
Illiterate	69 (72.6%)
Basic education	26 (27.4%)
**Previous malaria**	33 (34.7%)
**Nutritional status**	
Height-for-Age (Z score <-1)	40 (42.1%)
Height-for-Age (Z score <-2)	11(11.6%)
BMI-for-age (Z score <-1)	17 (17.9%)
BMI-for-age (Z score <-2)	3 (3,2%)
**Hemoglobin (g/dL)**	11.5 IQR [11–12.2]
**Anemia**	52 (54.7%)
**C-reactive protein**	4 IQR [3–5]
**Micronutrient deficiencies**	
Vitamin A	33 (35.9%)
Beta-carotene	58 (63.0%)
Zinc	56 (60.9%)
Iron	47 (51.1%)
**Intestinal parasitosis**	76(80.0%)
*Ascaris lumbricoides*	42(44.2%)
*Giardia lamblia*	37(39.0%)
*Entamoeba histolytica/dispar*	18(19.0%)
*Trichuris trichiura*	17(17.9%)
Hookworms	3 (3.2%)
*Enterobius vermicularis*	3 (3.2%)

Abbreviations: IQR: Inter-quartile rage; N: number; BMI: Body mass index.

Cut-off points for micronutrient deficiency: ≤10 μg/dL for vitamin A, ≤20 μg/dL for beta-carotene, ≤70 μg/dL for zinc and ≤70 μg/dL for iron. Anemia was defined as hemoglobin <12 g/dL (47).

### Impact of micronutrient deficiencies on malaria incidence

**Fig 2:**
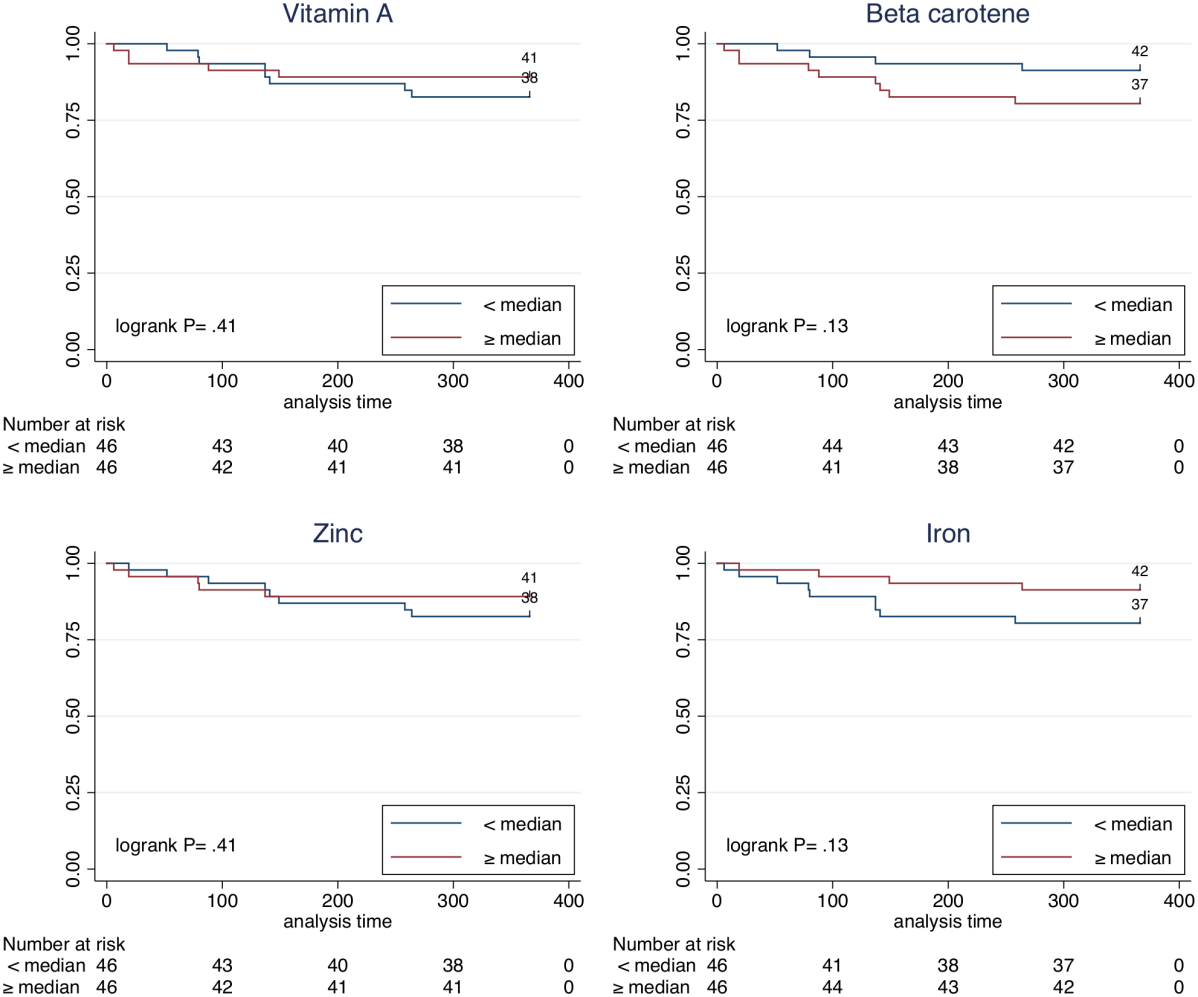
Survival analysis of time to first *Plasmodium vivax* malaria episode. Time to the first malaria episode was similar between participants with micronutrient levels above or below the median, for vitamin A, beta-carotene, zinc and iron.

We followed 95 children through passive surveillance for a year and detected 17 cases of malaria, 16 by *P*. *vivax* and 1 by *P*. *falciparum*. Three patients had *P*. *vivax* malaria twice during the observation period. In the time to *P*. *vivax* incidence analyses, baseline vitamin A serum levels were not associated with the outcome (Hazard ratio [HR]: 1.51; 95%confidence interval [CI]: 0.51–4.52), nor did beta-carotene (HR: 0.47; 95%CI: 0.16–1.42), or zinc (HR: 1.41; 95%CI: 0.43–4.57) (Table 2 and Fig 2). We found a slight trend towards an association of iron deficiency and malaria incidence (HR: 2.31; 95%CI: 0.71–7.51) and also found a similar trend for maternal illiteracy (HR: 4.81; 95%CI: 0.62–36.97), having had malaria (HR: 2.33; 95%CI: 0.78–6.94), and the number of intestinal parasites detected during the baseline evaluation (HR: 1.59; 95%CI: 0.89–2.85). Association strengths remained similar to crude results after adjustment for potential confounders (sex, age, maternal illiteracy and intestinal parasites).

**Table 2:** Univariate Cox Proportional Hazard analyses of baseline characteristics associated with time to malaria incidence in Careiro, Brazil, May 2010.

Variables	First year (*P*. *vivax* cases = 13)		
	Hazard ratio	p-value	95% CI
Age (years)	1.08	0.48	0.87–1.33
Male	0.76	0.62	0.25–2.25
Maternal Illiteracy	4.81	0.13	0.62–36.97
Previous malaria	2.33	0.13	0.78–6.94
Height-for-Age (Z score <-1)	1.17	0.78	0.39–3.48
BMI-for-age (Z score <-1)	1.42	0.59	0.39–5.17
Hemoglobin <12 mg/dL	0.87	0.83	0.26–2.90
Vitamin A deficiency	1.51	0.45	0.51–4.52
Beta-carotene deficiency	0.47	0.19	0.16–1.42
Zinc deficiency	1.41	0.57	0.43–4.57
Iron deficiency	2.31	0.16	0.71–7.51
Number of intestinal parasites	1.59	0.12	0.89–2.85

Abbreviations: *P*. *vivax*: *Plasmodium vivax*, CI: Confidence interval. BMI: Body mass index.

Cut-off points for micronutrient deficiency: ≤10 μg/dL for vitamin A, ≤20 μg/dL for beta-carotene, ≤70 μg/dL for zinc and ≤70 μg/dL for iron.

### Impact of malaria in micronutrients

The impact of having had malaria on micronutrient levels was assessed based on reevaluation conducted 1 year after the baseline assessment. Because 27 children had moved out of the study area by reevaluation time, the remaining 68 children were reassessed for micronutrient levels and these results were compared with baseline assessments to obtain the Δ for each micronutrient. Having had malaria was not associated with a Δ of any of the assessed micronutrients (Table 3). Adjustment for potential confounders (sex, age, maternal illiteracy and intestinal parasites) did not change these results substantially.

**Table 3:** Association between malaria during the observation period (May 2010–May 2011) and less than median micronutrient serum levels during the same period.

Below median	2010 median (μg/dL)	2011 median (μg/dL)	Malaria (N = 56)	No malaria (N = 12)	p-value
Delta (Δ) Vitamin A	21.0	20.0	29 (52.7%)	5 (38.4%)	0.54
Δ Beta-caroten*	25.8	19.3	25 (46.3%)	8 (66.7%)	0.34
Δ Iron*	70.0	69.8	27 (50.0%)	6 (46.2%)	0.80
Δ Zinc*	67.6	69.8	27 (50.9%)	6 (46.2%)	0.76

*P*-values calculated by Chi2 or Fisher’s exact test.

Cases that were evaluated both in 2010 and 2011 are shown.

*2 children lacked data for Δ serum level of beta-carotene and zinc, and 1 child for Δ serum level of iron.

## Discussion

We evaluated the bidirectional association of vivax malaria and micronutrient deficiencies and found no association of deficiencies of vitamin A, beta-carotene, zinc, or iron with malaria incidence. Incident malaria evaluated throughout a year was not associated with worsening of micronutrient deficiencies either. We reviewed the extant literature to compare our results with previous findings, mostly from *P*. *falciparum* endemic areas.

Vitamin A deficiency is found in 17.4% of children in Brazil [50, 51]. In our study, more than one third of children were vitamin A deficient and about two thirds had deficiency of the vitamin A precursor beta-carotene. Such results highlight the profound differences between the underdeveloped areas in the Brazilian Amazon and the rest of to the country. In addition, the data shows a nutritional paradox reflected in this area with abundance of fruits rich in precursors of vitamin A where inhabitants present important deficiencies, especially children from a lower socio-economical status [52–54]. Most children had also deficiencies in zinc (61%) and iron (51%) at proportions comparable to the sub-Saharan region [55]. These findings should be considered by the Ministry of Health to implement educational strategies to increase the intake of regional foods rich in vitamin A precursors and minerals, such as zinc and iron.

Our results did not support an association of vitamin A levels at baseline and incidence of *P*. *vivax* malaria in the Brazilian Amazon. Previous studies, mostly in *P*. *falciparum* had shown an association of vitamin A levels with severity [17, 56] and mortality [57, 58], prompting several randomized controlled trials using vitamin A supplementation as a means to improve malaria outcomes [59, 60]. However most studies showing an association between micronutrients and malaria involved cases of *P*. *falciparum* malaria. One published study in an area where *P*. *vivax* is predominant assessed hemoglobin levels by day 30 and found no difference between vitamin A supplementation and the control group [18], in agreement with our findings

We did not find an association between zinc deficiency and malaria incidence. Interestingly that studies have addressed the role of zinc supplementation in malaria outcomes have shown dissimilar results. Some studies found a protective effect of zinc supplementation on the number of malaria related clinic visits in Gambia [61] and rates of malaria incidence in Papua New Guinea [4], while a study in Burkina Faso showed no association [62]. All these studies had a predominance of *P*. *falciparum* malaria and results may differ in *P*. *vivax* endemic areas. Our results are similar to a study that found no association of zinc supplementation and *P*. *vivax* outcomes in Peru where *P*. *vivax* also predominates [63].

We found a slight trend towards an association of iron deficiency and malaria incidence and a similar trend for maternal illiteracy, having had malaria and the number of intestinal parasites detected during the baseline evaluation. These parameters are likely interrelated and may serve as markers of a lower socio-economical status: living in areas with the poorest hygienic conditions may result in an increased risk of acquiring malaria. This trend, although not statistically significant, may go against previous studies that found a possible protective effect of lower iron levels on *P*. *falciparum* incidence [23]. An association between iron supplementation and malaria mortality was reported in Zanzibar [64]. In a study in the Peruvian Amazon with similar epidemiologic characteristics to our study group [63], the authors detected an increased risk of malaria incidence after iron supplementation. A previous study by our group assessed 168 children in the same study location and found low levels of meat, fish or poultry consumption [54, 65]. These results explain the high prevalence of zinc and Iron deficiencies in our study population as these micronutrients come mostly from the consumption of animal products. Children are not offered these products as a result of these being considered “reimosos”, a tightly held food taboo in the Amazon region [54, 66–68].

We also assessed whether patients having malaria during the 1 year follow up would have a downtrend in their micronutrient serum levels by comparing the measurements from 2010 and 2011. We did not find a statistically significant association of malaria incidence and Δ of any of the micronutrients. Previous studies have shown that malaria incidence may reduce the ingestion of micronutrients [69] and their absorption [70]. These studies were conducted in *P*. *falciparum* endemic areas. Our study does not support the same effect by *P*. *vivax*.

Our findings should be interpreted in the context of some limitations. We report the experience of one municipality in the Brazilian Amazon and our findings may not be generalizable to other settings. Our sample size and, especially, the low number of events, could have prevented us from detecting existing associations a larger population would be able to. Unmeasured patient, socio-economical and administrative factors may affect malaria incidence and micronutrient levels. We were not able to assess dietary patterns, and although there is evidence of homogeneous habits within the study population [7, 65], there may be important individual variations that may have an impact on the nutritional status of children. We performed passive surveillance for malaria incidence, and possibly more cases and asymptomatic ones could have been detected if we had implemented an active surveillance system. Despite these limitations, our study advances the knowledge of nutritional status and *P*. *vivax* malaria in several ways. We found a high prevalence of micronutrient deficiencies and intestinal parasitosis in the study area.

In conclusion, we were not able to observe an association between serum micronutrient levels and malaria incidence, although we found a trend towards an association between iron deficiency and malaria incidence. Iron deficiency may be a marker of poorer socio-economical status and higher malaria risk as a result. Further studies assessing micronutrient levels in *P*. *vivax* endemic areas in larger populations are needed to validate our results.
